# Development and validation of a novel nomogram model for identifying risk of prolonged length of stay among patients receiving free vascularized flap reconstruction of head and neck cancer

**DOI:** 10.3389/fonc.2024.1345766

**Published:** 2024-05-02

**Authors:** Chengli Wang, Liling Lin, Jiayao Wu, Ganglan Fu, Zhongqi Liu, Minghui Cao

**Affiliations:** ^1^ Department of Anesthesiology, Sun Yat-sen Memorial Hospital, Sun Yat-sen University, Guangzhou, China; ^2^ Guangdong Provincial Key Laboratory of Malignant Tumor Epigenetics and Gene, Regulation, Sun Yat-sen Memorial Hospital, Sun Yat-sen University, Guangzhou, China; ^3^ Shenshan Medical Center, Memorial Hospital of Sun Yat-sen University, Shanwei, China; ^4^ Department of Anesthesiology, Guangdong Women and Children Hospital, Guangzhou, China

**Keywords:** nomogram, length of hospital stay, free vascularized flap, reconstruction, head and neck cancer

## Abstract

**Background:**

The aim of the present study was to build and internally validate a nomogram model for predicting prolonged length of stay (PLOS) among patients receiving free vascularized flap reconstruction of head and neck cancer (HNC).

**Methods:**

A retrospective clinical study was performed at a single center, examining patients receiving free vascularized flap reconstruction of HNC from January 2011 to January 2019. The variables were obtained from the electronic information system. The primary outcome measure was PLOS. Univariate and multivariate analyses were used to find risk factors for predicting PLOS. A model was then built according to multivariate results. Internal validation was implemented via 1000 bootstrap samples.

**Results:**

The study included 1047 patients, and the median length of stay (LOS) was 13.00 (11.00, 16.00) days. Multivariate analysis showed that flap types ((radial forearm free flap (odds ratio [OR] = 2.238; 95% CI, 1.403-3.569; P = 0.001), free fibula flap (OR = 3.319; 95% CI, 2.019-4.882; P < 0.001)), duration of surgery (OR = 1.002; 95% CI, 1.001-1.003; P = 0.004), postoperative complications (OR = 0.205; 95% CI, 0.129-0.325; P = P < 0.001) and unplanned reoperation (OR = 0.303; 95% CI, 0.140-0.653; P = 0.002) were associated with PLOS. In addition to these variables, blood transfusion was comprised in the model. The AUC of the model was 0.78 (95% CI, 0.711–0.849) and 0.725 (95% CI, 0.605–0.845) in the primary and internal validation cohorts, respectively. The DCA revealed the clinical utility of the current model when making intervention decisions within the PLOS possibility threshold range of 0.2-0.8.

**Conclusions:**

Our study developed a nomogram that exhibits a commendable level of accuracy, thereby aiding clinicians in assessing the risk of PLOS among patients receiving free vascularized flap reconstruction for HNC.

## 1 Introduction

Head and neck cancer (HNC) ranks seventh among the prevalent malignancies globally (1), with surgical resection being the primary and widely employed treatment modality. Nevertheless, surgical resection would lead to head and neck defects (HND) that may impede the functionality or anatomical integrity of HNC patients. Consequently, it is customary to undertake free flap transplantation concurrently with tumor removal to address and rectify HND. The intricate nature of surgical procedures such as primary lesion resection, flap dissociation and transfer repair,: and vascular anastomosis necessitates a longer period for postoperative functional recovery, leading to extended hospital stays for patients undergoing these procedures. As regards HNC patients undergoing free vascularized flap reconstruction, the LOS can vary significantly, ranging from a few days to several weeks or even months (2–4). This variability in LOS plays a crucial role in patient rehabilitation and the determination of appropriate multimodal adjuvant therapy. In addition, prolonged LOS (PLOS) may result in more hospitalization fees.

In the decades, the idea of Enhanced Recovery After Surgery (ERAS) was widely popularized, leading surgeons and anesthesiologists involved in the perioperative period to increasingly recognize the significance of implementing ERAS. The core objective of this concept is to better the preoperative condition of patients, guarantee their safety, reduce the perioperative adverse stimulation and the length of hospital stay (LOS), and expedite the recovery process for surgical patients (5, 6). In the case of patients with HNC, promptly identify risk factors predicting PLOS and effectively reduce hospitalization time within the framework of ERAS.

Numerous risk factors have been identified as being related to PLOS in HNC patients including preoperative malnutrition, excessive alcohol use, diabetes mellitus, American Society of Anesthesiologists Classification (ASA), preoperative anemia, perioperative blood transfusion, insufficient fluid given rate during 24 h, and anesthesia or surgery time (7–10). However, no study included all types of free flaps and encompass a comprehensive range of variables. People closely supervised LOS as a prominent outcome measurement, serving as hospital quality control indicators that are associated with patient prognosis and care costs. PLOS exhibits correlations with various perioperative adverse outcomes, including an elevated risk of hospital acquired infections and deep vein thrombosis. The timely identification of risk factors contributing to PLOS assumes particular significance in reducing hospitalization expenses and enhancing the patient’s rehabilitation journey.

Clinicians often visualize representation of results using nomograms. Nevertheless, there is currently no available nomogram specifically designed to predict the risk of PLOS among patients who underwent free vascularized flap reconstruction of HNC. We hypothesized that perioperative surgical and anesthesia-related variables could be used to predict the risk of PLOS in these patients. Consequently, the purpose of the present study was to build and validate a novel prediction nomogram model that can accurately estimate the risk of PLOS in this specific patient population.

## 2 Patients and methods

### 2.1 Study sample

The current retrospective investigation was conducted at a single center, encompassing all individuals who underwent resection of HNC and subsequent reconstruction using different types of free flaps. The study period spanned from January 2011 to January 2019, and the data used in the current study were partially obtained from a previously published article authored by our team (11). The research was granted approval by the Medical Ethical Committee of Sun Yat-sen Memorial Hospital, Sun Yat-sen University. In consideration of the retrospective nature of the study, an exemption of informed consent was sought.

### 2.2 Inclusion and exclusion criteria

This study contained adult patients who underwent HNC resection, with subsequent reconstruction of HND using different types of free flaps such as anterolateral thigh free flap (ALTFF), fibula free flap (FFF), radial forearm free flap (RFFF), or calf fasciocutaneous free flap (CFFF). Exclusion criteria encompassed: (1) patients with incomplete electronic medical records; (2) patients who received HND reconstruction through pedicled flaps.

### 2.3 Variables

Perioperative predictive variables were gathered based on our previously published studies (10–12). We collected three categories of perioperative variables. The initial category consisted of general clinical traits such as gender, age, weight, comorbidities, smoking habits, primary lesions, and chemoradiotherapy. The second category encompassed hemodynamic related variables, containing perioperative plasma concentrations of albumin (Alb) and hemoglobin (Hb), infusion rate and total volume of colloid and crystalloid both among the surgery and postoperative 24 h, transfusion, amount of bleeding, and intraoperative urine output. We divided urine output into three grades. The liquid infusion rate was standardized based on the weight during the surgery and over the course of 24 hours. The last category encompasses anesthetic and surgical factors, such as ASA grade, flap types (RFFF, FFF, ALTFF, and CFFF), duration of surgery, unplanned reoperation, and postoperative complications. We defined unplanned reoperation as the occurrence of an operation within 30 days following the initial surgery. The criteria for inclusion of postoperative complications encompass those that manifest during hospitalization and are categorized as Grade II or higher according to the Clavien-Dindo classification system. These complications encompass both surgical complications (such as total or partial necrosis, thromboembolism, bleeding, dehiscence, fistula, or flap infection) and medical complications (including pneumonia, pneumothorax, hydrothorax, pulmonary embolism, heart failure, atrial fibrillation, deep venous thrombosis, cerebral stroke, ileus, and ketoacidosis).

The LOS was defined as the main outcome measure, recorded as interval between the day of surgical procedure and the patient’s leaving hospital day. The average LOS was represented by the median due to the non-normal distribution of the LOS data. Subsequently, LOS values exceeding the median were categorized as prolonged LOS (PLOS), while LOS values equal to or less than the median were categorized as non-PLOS. In view of the median LOS, we divided all the participants into two groups: “non-PLOS” (≤median) and “PLOS” (>median).

### 2.4 Univariate and multivariate analyses

The primary and validation cohorts were evaluated for the univariable association of general clinical characteristics, hemodynamic variables, anesthetic and surgical variables. Risk factors predicting PLOS was identified by univariate and multivariate analyses by comparing the PLOS and non-PLOS groups. The selection of characteristics for the multivariable comparison was based on collinearity diagnostics.

### 2.5 Establishment and verification of the novel nomogram

To develop a novel predictive model, backward stepwise multivariable logistic regression analysis was conducted (13, 14). To facilitate the prediction of individual PLOS probability, a novel nomogram was devised utilizing the developed prediction model. The nomogram underwent bootstrapping validation to ascertain adjusted AUC within the initial cohort. Subsequently, the multivariable logistic regression model derived from the initial cohort was employed to evaluate all patients within the verification cohort. Logistic regression analysis was subsequently conducted in the verification cohort, incorporating the total scores as a covariate. Receiver operating characteristic (ROC) curve, C-index, and calibration curve analyses were conducted in both the initial and verification cohorts.

### 2.6 Clinical benefit evaluation

Decision curve analysis (DCA) (15) was conducted to assess the utility of this nomogram in predicting PLOS.

### 2.7 Statistical analysis

Continuous data were compared by the student t-test or the Mann-Whitney U test, according to distribution type of data. Categorical data were compared by either the chi-square test or Fisher’s exact test according to data frequencies. Univariable and multivariable logistic regression analysis were employed using the IBM SPSS software (version 25.0; SPSS Inc, Chicago, IL) to find risk factors predicting PLOS. The development, validation of the novel nomogram, and decision curve analysis (DCA) were conducted using R software (version 3.0.4; http://www.Rproject.org). The “rms” and “rmda” packages of R were utilized for the study. Variables with a significant level of *P* < 0.05 were considered statistical significance.

## 3 Results

### 3.1 Study sample and general clinical characteristics

A comprehensive sample of 1059 patients who received HNC resection and subsequent reconstruction of various free vascularized flaps, such as ALTFF, FFF, RFFF, or CFFF, for the repair of HNC between January 2011 and January 2019, was initially gathered. After excluding 12 patients with incomplete electronic medical records, finally, 1047 patients were included in the present study. Wherein, 357 patients received ALTFF reconstruction for HND repair, 354 patients received FFF reconstruction, 312 patients received RFFF reconstruction, and only 24 patients received CFFF reconstruction. The selection of a free flap by HNC surgeons was determined by the extent and location of the HND. The median LOS was recorded as 13.00 (11.00, 16.00) days. Based on this median LOS, patients were categorized into two different groups: the non-PLOS group (n = 599, LOS ≤ 13 days) and the PLOS group (n = 448, LOS > 13 days). To construct a predictive model, 733 patients were then randomly assigned to the initial cohort, while the other 314 patients were randomly distributed to the verification cohort. The general clinical characteristics of patients within the initial and verification cohorts can be found in **Table 1**. The two cohorts were comparable.

**Table 1:** Patient characteristics in the primary and validation cohorts.

	Primary Cohort(n=733)	Validation Cohort(n=314)	P value
Flap types			0.868
RFFF	220 (30.0)	92 (92.3)	
CFFF	16 (2.2)	8 (2.5)	
FFF	252 (34.4)	102 (32.5)	
ALTFF	245 (33.4	112 (35.7)	
Age	55 (46, 63)	57 (47, 65)	0.063
Weight	58 (52, 65)	58 (52, 66)	0.54
Preoperative Hb	133 (120, 144)	133 (121, 143)	0.812
Preoperative ALB	40.2 (36.8, 42.8)	39.5 (36.7, 42.6)	0.114
Postoperative Hb	107 (97, 120)	108 (97, 119)	0.925
Postoperative ALB	29.1 (25.8, 31.6)	29.4 (25.8, 31.6)	0.443
Duration of surgery	445 (335, 500)	445 (325, 550)	0.55
Blood loss	400 (300, 500)	400 (300, 500)	0.416
Liquid infusion over 24 h	5200 (4600, 5850)	5150 (4500, 5750)	0.225
Crystal infusion over 24 h	4250 (3550, 4750)	4050 (3550, 4700)	0.109
Colloid infusion over 24 h	1000 (600, 1200)	1000 (550, 1250)	0.679
Gender (male)	487 (66.4)	208 (66.2)	0.951
ASA			0.574
2	406 (55.4)	168 (53.5)	
3	327 (44.6)	146 (46.5)	
Smoke	244 (33.3)	112 (35.7)	0.456
Comorbidities			0.376
none	562 (76.7)	233 (71.0)	
hypertension	96 (13.1)	50 (15.9)	
diabetes	26 (3.5)	11 (3.5)	
hypertension and diabetes	40 (5.5)	26 (8.3)	
CAD	4 (0.5)	3 (1.0)	
COPD	3 (0.4)	1 (0.3)	
CKD	2 (0.3)	0 (0.0)	
Chemoradiotherapy	105 (14.3)	40 (12.7)	0.496
Primary disease			0.648
tongue cancer	485 (66.2)	207 (65.9)	
carcinoma of mouth floor	121 (16.5)	48 (15.3)	
gingival cancer	46 (6.3)	20 (6.4)	
Buccal carcinoma	29 (4.0)	19 (6.1)	
oropharyngeal cancer	52 (7.1)	20 (6.4)	
Blood transfusion	245 (33.4)	100 (31.8)	0.619
Urine output			0.89
low	11 (1.5)	6 (1.9)	
medium	264 (36.0)	113 (36.0)	
high	458 (62.5)	195 (62.1)	
Intraoperative crystalloid infusion rate			0.12
low	415 (56.6)	194 (61.8)	
high	318 (43.4)	120 (38.2)	
Intraoperative colloid infusion rate			0.963
low	321 (43.8)	138 (43.9)	
high	412 (56.2)	176 (56.1)	
Crystalloid infusion rate over 24 h			0.143
low	377 (51.4)	177 (56.4)	
high	356 (48.6)	137 (43.6)	
Colloid infusion rate over 24 h			0.741
low	342 (46.7)	150 (47.8)	
high	391 (53.3)	164 (52.2)	
Liquid infusion rate over 24 h			0.053
low	370 (50.5)	179 (57.0)	
high	363 (49.5)	135 (43.0)	
Postoperative complications	206 (28.1)	73 (23.3)	0.11
Unplanned reoperation	88 (12.0)	27 (8.6)	0.106

RFFF, radial forearm free flap; CFFF, calf fasciocutaneous free flap; FFF, fibula free flap; ALTFF, anterolateral thigh free flap; Hb, hemoglobin; ALB, albumin; ASA, American Society of Anesthesiologists; CAD, Coronary artery diseases; COPD, chronic obstructive pulmonary disease; CKD, chronic kidney disease.

### 3.2 Univariate and multivariate logistic analysis of the initial cohort

The statistical analyses comparing the PLOS and non-PLOS groups within the initial cohort, were displayed in **Table 2**. Univariate logistic analyses revealed significant differences between the two groups in various factors, including flap types, postoperative Alb levels, duration of surgery, blood loss, chemoradiotherapy, transfusion, intraoperative infusion rate of crystalloid, unplanned reoperation and postoperative complications (**Table 2**). In the context of multivariate logistic analysis, it was determined that flap types, duration of surgery, unplanned reoperation and postoperative complications were significant associated with the possibility of PLOS among patients receiving free vascularized flap reconstruction for the HNC (**Table 2**).

**Table 2:** Univariate and multivariate comparisons between the PLOS and non-PLOS Groups in the primary cohort.

Characteristics	Non-PLOS (n=412)	PLOS (n=321)	univariate	multivariate	OR	95%CI
Flap types			<0.001	<0.001		
RFFF	134 (32.5)	86 (26.8)		0.001	2.238	1.403 to 3.569
CFFF	8 (1.9)	8 (2.5)		0.061	3.047	0.950 to 9.775
FFF	102 (24.8)	150 (46.7)		<0.001	3.139	2.019 to 4.882
ALTFF	168 (40.8)	77 (24.0)				
Age	54 (45, 64)	55 (46, 63)	0.742			
Weight	58 (52, 65)	57 (52, 65)	0.362			
Preoperative Hb	133 (120, 145)	132 (120, 144)	0.532			
Preoperative ALB	40.2 (37.2, 42.8)	39.9 (36.6, 42.8)	0.241			
Postoperative Hb	108 (97, 121)	106 (96, 118)	0.163	0.37	1.006	0.993 to 1.019
Postoperative ALB	29.5 (26.4, 31.6)	28.2 (24.7, 31.7)	0.011	0.53	0.986	0.945 to 1.030
Duration of surgery	435 (330, 530)	465 (343, 580)	0.008	0.048	1.001	1.000 to 1.003
Blood loss	300 (200, 500)	400 (300, 500)	<0.001	0.392	1	0.999 to 1.001
Liquid infusion over 24 h	5200 (4650, 5788)	5250 (4500, 5910)	0.978			
Crystal infusion over 24 h	4250 (3650, 4750)	4200 (3400, 4835)	0.716			
Colloid infusion over 24 h	1000 (600, 1200)	1000 (600, 1500)	0.529			
Gender (male)	274 (66.5)	213 (66.4)	0.966			
ASA			0.077	0.946	0.988	0.692 to 1.411
2	240 (58.3)	166 (51.7)				
3	172 (41.7)	155 (48.3)				
Smoke	141 (34.2)	103 (32.1)	0.543			
comorbidities			0.571			
none	322 (78.2)	240 (74.8)				
hypertension	48 (11.7)	48 (15.0)				
diabetes	15 (3.6)	11 (3.4)				
hypertension and diabetes	21 (5.1)	19 (5.9)				
CAD	3 (0.7)	1 (0.3)				
COPD	1 (0.2)	2 (0.6)				
CKD	2 (0.5)	0 (0.0)				
Chemoradiotherapy	48 (11.7)	57 (17.8)	0.019	0.728	0.911	0.539 to 1.539
Primary disease			0.485			
tongue cancer	276 (67.0)	209 (65.1)				
carcinoma of mouth floor	63 (15.3)	58 (18.1)				
gingival cancer	23 (5.6)	23 (7.2)				
buccal carcinoma	16 (3.9)	13 (4.0)				
oropharyngeal cancer	34 (8.3)	18 (5.6)				
Blood transfusion	116 (28.2)	129 (40.2)	0.001	0.346	0.814	0.530 to 1.250
Urine output			0.386			
low	4 (1.0)	7 (2.2)				
medium	147 (35.7)	117 (36.4)				
high	261 (63.3)	197 (61.4)				
Intraoperative crystalloid infusion rate			0.022	0.436	1.167	0.791 to 1.721
low	218 (52.9)	197 (61.4)				
high	194 (47.1)	124 (38.6)				
Intraoperative colloid infusion rate			0.256			
low	188 (45.6)	133 (41.4)				
high	224 (54.4)	188 (58.6)				
Crystalloid infusion rate over 24 h			0.448			
low	217 (52.7)	160 (49.8)				
high	195 (47.3)	161 (50.2)				
Colloid infusion rate over 24 h			0.168	0.25	1.25	0.855 to 1.828
low	183 (44.4)	159 (49.5)				
high	229 (55.6)	162 (50.5)				
Liquid infusion rate over 24 h			0.369			
low	214 (51.9)	156 (48.6)				
high	198 (48.1)	165 (51.4)				
Postoperative complications	48 (11.7)	158 (49.2)	<0.001	<0.001	0.208	0.131 to 0.332
Unplanned reoperation	12 (2.9)	76 (23.7)	<0.001	0.001	0.286	0.132 to 0.619

RFFF, radial forearm free flap; CFFF, calf fasciocutaneous free flap; FFF, fibula free flap; ALTFF, anterolateral thigh free flap; Hb, hemoglobin; ALB, albumin; ASA, American Society of Anesthesiologists; CAD, Coronary artery diseases; COPD, chronic obstructive pulmonary disease; CKD, chronic kidney disease; OR, odds ration; CI, confidence interval.

### 3.3 Establishment and verification of the novel nomogram

**Figure 1:**
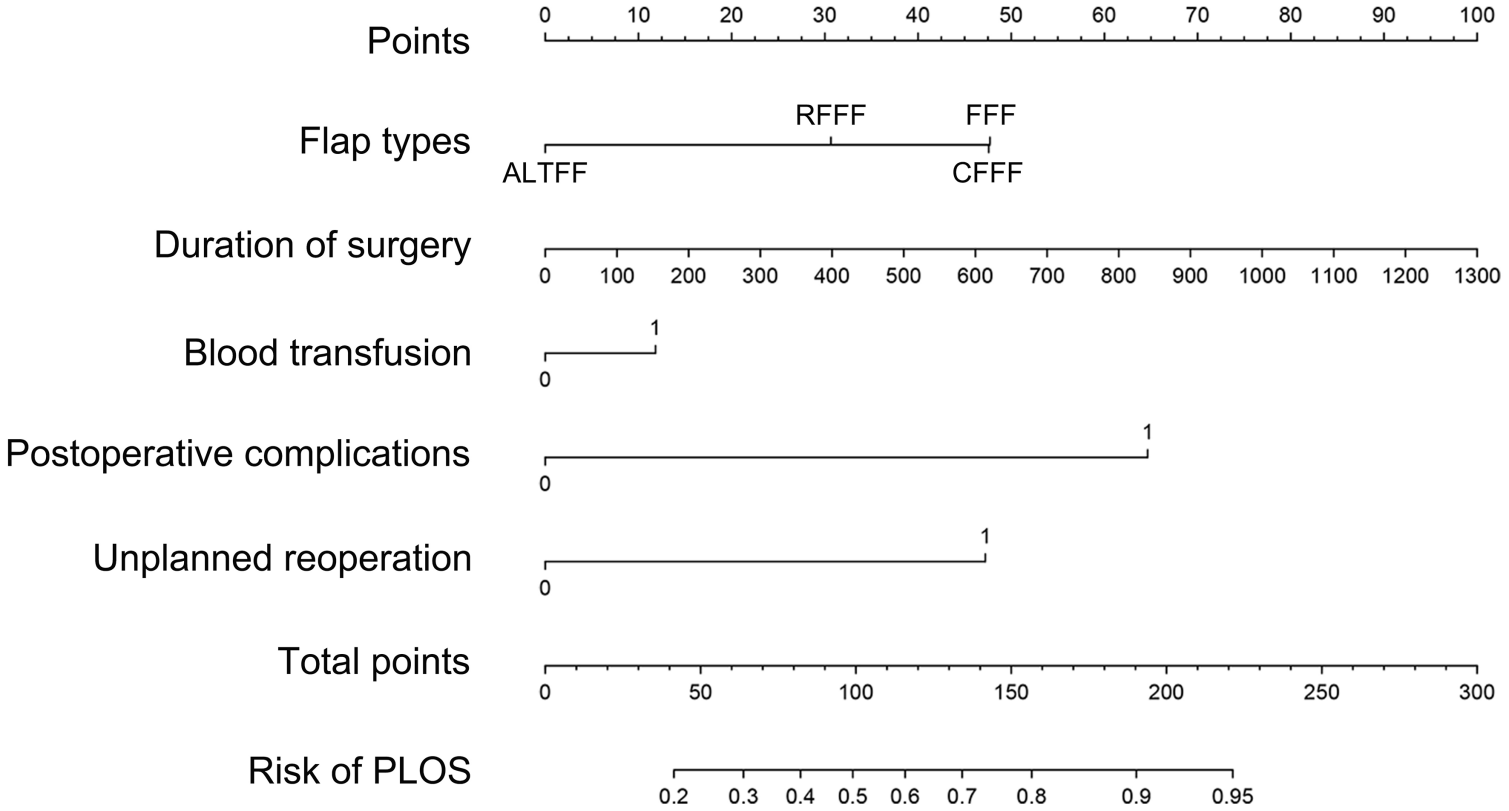
Nomogram derived from backward stepwise logistic regression analysis.

**Figure 2:**
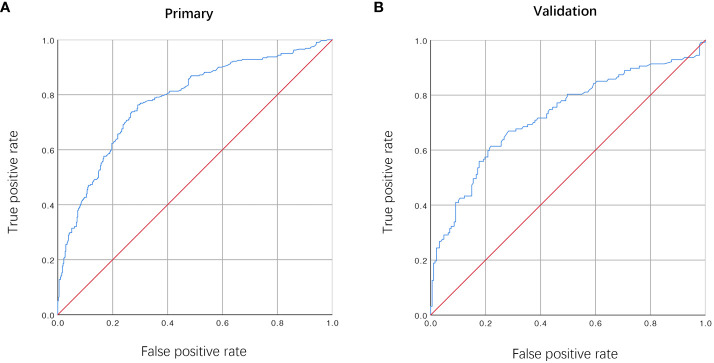
The ROC curves of the primary **(A)** and validation cohort **(B)**.

**Figure 3:**
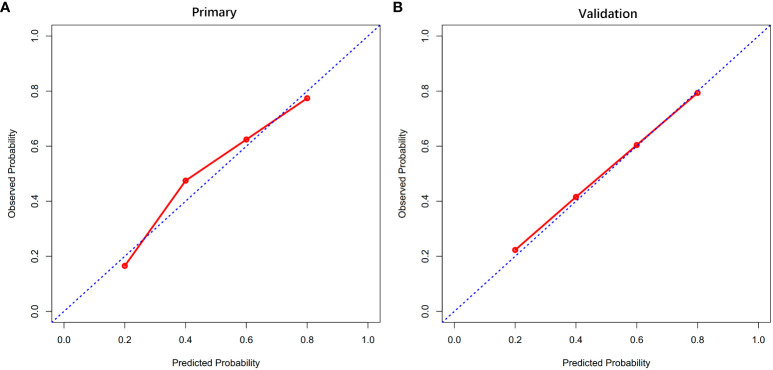
Calibration curves of the primary **(A)** and validation cohort **(B)**.

The findings from the multivariate analysis further support the identification of flap types, duration of surgery, unplanned reoperation and postoperative complications as potential risk variables for PLOS (**Table 3**). A predictive model incorporating the aforementioned independent risk variables and blood transfusion for PLOS was constructed and visualized as a nomogram (**Figure 1**). The ROC curves for both the primary and verification cohorts were presented in **Figure 2**. The C-index of the prediction model was 0.78 (95% confidence interval [CI], 0.711–0.849) and 0.725 (95% CI, 0.605–0.845) in the primary and verification cohorts, respectively (**Table 3**). The calibration curves demonstrated that the novel model exhibited good agreement with the observed incidence of PLOS in both the initial and verification cohorts (**Figure 3**).

**Table 3:** Risk factors for PLOS derived from backward stepwise logistic regression analysis.

	multivariate	OR	95%CI
Flap types	<0.001		
RFFF	0.001	2.216	1.413 to 3.475
CFFF	0.051	3.159	0.994 to 10.041
FFF	<0.001	3.221	2.110 to 4.918
ALTFF			
Duration of surgery	0.004	1.002	1.001 to 1.003
Blood transfusion	0.116	0.737	0.503 to 1.079
Postoperative complications	<0.001	0.205	0.129 to 0.325
Unplanned reoperation	0.002	0.303	0.140 to 0.653
C for primary		0.78	0.711 to 0.849
C for validation		0.725	0.605 to 0.845

RFFF, radial forearm free flap; CFFF, calf fasciocutaneous free flap; FFF, fibula free flap; ALTFF, anterolateral thigh free flap; OR, odds ration; CI, confidence interval.

### 3.4 Clinical benefit of the novel nomogram

**Figure 4:**
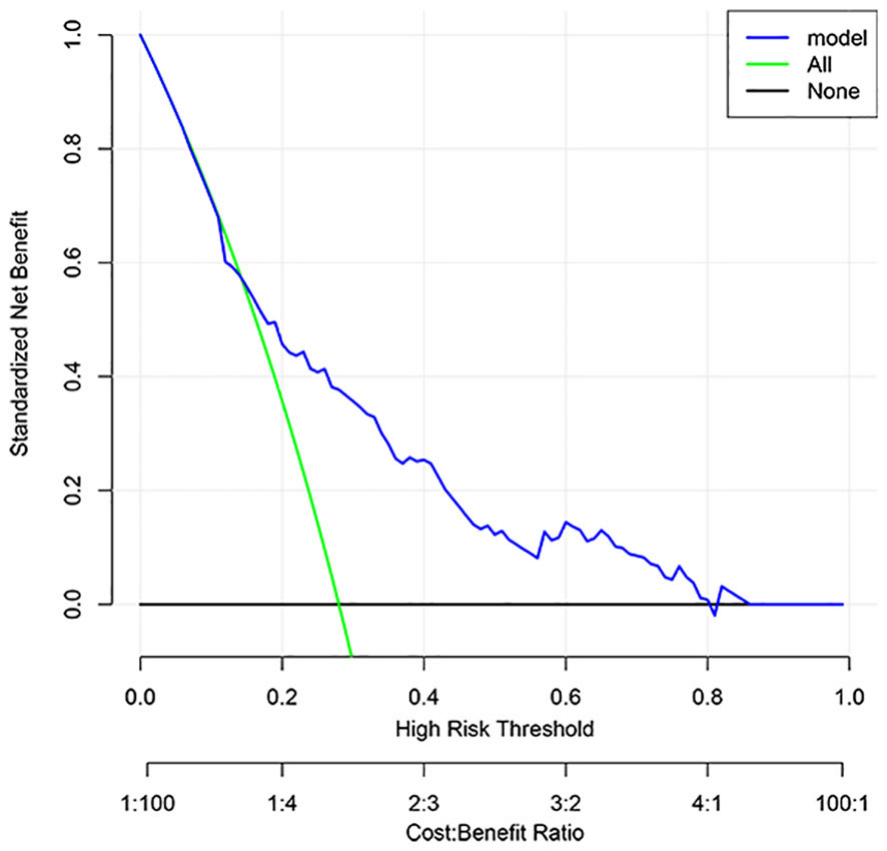
The decision curve analysis of the novel nomogram.

The DCA result for the PLOS nomogram was showed in **Figure 4** which revealed that if the threshold probability was set between 20% and 80%, the utilization of the current innovative nomogram yielded greater benefits compared to both the treat-all scheme and the treat-none scheme.

## 4 Discussion

In contemporary times, the ERAS gained increasing traction and has been progressively integrated into clinical settings. The LOS for surgical patients holds considerable importance for both patients and medical establishments, and it is influenced by numerous perioperative factors. The LOS primarily serves as an indicator of patients’ postoperative recovery rate, and the primary objective of ERAS is to expedite the reduction of LOS for patients. In this particular context, we have established and verified a concise prediction nomogram model to assess the possibility of PLOS among patients who received HNC resection and subsequent free vascularized flap reconstruction for HND. Our newly devised nomogram model incorporates a mere five readily accessible variables, rendering it comprehensible and convenient for head and neck surgeons to employ in facilitating more precise clinical decision-making.

### 4.1 Flap types

In the present study, the identification of flap types as an autonomous risk factor for PLOS was observed. Typically, head and neck surgeons select the appropriate free flap for HND repair based on factors such as the primary lesion location, patient’s overall health, and the size of the HNC. Nevertheless, the impact of different free flap types on the short-term prognosis of patients who received HNC resection and subsequent free vascularized flap reconstruction for HND remains a subject of controversy. Lindeborg et al. (16) performed a retrospective clinical study with a limited sample size and found no significant association between flap type and PLOS. However, it is important to note that the majority of free flaps included in their study were RFFF, while the sample size for other free flap types was relatively small. Therefore, further investigation is necessary to find the association between flap type and PLOS. In our study, we included a larger sample size of various flap types. The findings from the backward stepwise logistic regression analysis indicated that patients who underwent FFF had a 3.221-fold increasing risk of PLOS compared with those who underwent ALTFF, while patients who underwent RFFF had a 2.216-fold increased risk. However, it should be noted that CFFF did not show a significant association with PLOS, possibly by reason of the limited sample size. According to our previous study (12), we found that flap type was a potential risk variable for predicting postoperative complications in HNC free vascularized flap reconstruction, and patients who experienced complications had a relatively longer duration of hospital stay compared to those unexperienced complications.

Hence, it is imperative for HNC surgeons to thoroughly evaluate patients’ overall condition, as well as the size and placement of the HND, in order to make an informed decision regarding the most suitable free flap for repair. This approach aims to minimize the likelihood of complications and expedite the LOS.

### 4.2 Duration of surgery

The relationship between extended operation duration and length of hospital stay remains a topic of debate. Previous research has established a correlation between extended surgical duration and heightened occurrence of postoperative surgical complications and PLOS among patients who received free vascularized flap reconstruction for HNC (17, 18). Nevertheless, Lindeborg et al. (16) have reported that the duration of surgery did not exhibit a significant correlation with PLOS. According to their perspective, advancements in free vascularized flap surgical techniques and decreasing operative times may have diminished the significance of surgical duration as a prominent risk variable for complications. In recent years, despite the significant advancements in surgical technology and equipment in China, the scarcity of head and neck surgeons has hindered the ability of many patients to undergo simultaneous resection of primary lesions and free flap reconstruction. Consequently, this has led to a prolonged duration of surgery, potentially exacerbating the stress response and increasing the likelihood of postoperative complications and PLOS. The findings of the current study indicated that the duration of surgery independently contributes to the risk of PLOS. Hence, to adhere to ERAS guidelines, it is recommended that the procedure be conducted concurrently by two distinct teams of head and neck surgeons. One team should focus on the primary lesion resection and microvascular anastomosis, while the other team should concentrate on the dissection and liberation of the flap. This approach aims to expedite patient recovery and reduce hospitalization duration.

### 4.3 Postoperative complications and unplanned reoperation

Patients who undergo HNC resection and free vascularized flap reconstruction are frequently susceptible to an increased risk of postoperative infection. This is primarily attributed to the presence of multiple wound areas, a relatively unclean surgical site, underlying comorbidities, and an extended duration of surgery (19). Notably, surgical site infections and wound complications, including fistula or breakdown, have been found to be significantly associated with PLOS. A study revealed that surgical site infections can occur in a substantial proportion of head and neck patients, ranging from 22% to 39%, even when antibiotic prophylaxis is administered prior to surgery (20). Additional studies have not only identified surgical site infection as a potential risk variable for PLOS, but have also shown that it increases the likelihood of readmission within 30 days (17, 21). Furthermore, it has been demonstrated that postoperative non-wound infections, such as pneumonia, which are common but preventable complications, are also associated with PLOS in HNC patients. In general, the need for unplanned reoperation indicates the occurrence of significant surgical complications (20). Previous research has shown that unplanned reoperation not only results in increased hospitalization costs and additional strain on medical and social resources, but also leads to psychological and physical distress in patients with HNC (22, 23). In this study, the results of multivariate analysis revealed that unplanned reoperation and postoperative complications were identified as potential risk variables for PLOS.

### 4.4 Blood transfusion

Previous studies have showed that blood transfusion was a potential risk variable for PLOS and increased rates of wound infection among patients received free vascularized flap reconstruction of HND (8, 24). However, in the current study, blood transfusion was determined to be significantly correlated with PLOS in univariate comparison, but did not show an association with PLOS in multivariate analysis. The potential explanation for this discrepancy lies in the differing definitions of the blood transfusion variable between our study and previous research. Specifically, while prior studies treated blood transfusion as a continuous variable measured in units of red blood cells or plasma, our study defined it as a binary variable denoting either a positive or negative occurrence.

In summary, we have devised a prognostic nomogram that may aid in the timely detection of patients with a heightened susceptibility to PLOS and facilitate the prompt implementation of interventions among individuals who underwent free vascularized flap reconstruction of the HND.

## 5 Limitations

Firstly, the limitations of our study stem from the inherent nature of its retrospective design. Secondly, the electronic medical record did not capture other potential confounding factors such as postoperative delirium and others that could potentially influence the occurrence of PLOS. Thirdly, while the sample size was not insignificant, it was exclusively obtained from a solitary tertiary hospital, necessitating external validation among a more diverse population of HNC patients.

## 6 Conclusion

In this study, we have successfully devised and verified a pioneering model that exhibits superior predictive efficacy. The nomogram model developed in this study offers valuable guidance for physicians in the selection of appropriate free flap types and the decision of whether to administer blood transfusions during surgical procedures. This model aims to assist HNC surgeons in assessing the likelihood of PLOS in patients who received free vascularized flap reconstruction for HND. By accurately estimating the risk of PLOS on an individual basis, head and neck surgeons can effectively reduce LOS and implement ERAS protocols for HNC patients, thereby facilitating more precise and personalized therapeutic interventions.

## Data availability statement

The raw data supporting the conclusions of this article will be made available by the authors, without undue reservation.

## Ethics statement

The studies involving humans were approved by Medical Ethical Committee of Sun Yat-sen Memorial Hospital, Sun Yat-sen University. The studies were conducted in accordance with the local legislation and institutional requirements. The ethics committee/institutional review board waived the requirement of written informed consent for participation from the participants or the participants’ legal guardians/next of kin because the nature of the present study was a retrospective study.

## Author contributions

CW: Data curation, Writing – original draft. LL: Writing – original draft. JW: Writing – original draft. GF: Data curation, Writing – original draft. ZL: Methodology, Writing – review & editing. MC: Funding acquisition, Writing – review & editing.
